# Bilateral Ultrasound-Guided Transversus Thoracic Plane Block as Part of a Multimodal Analgesic Plan in Two Rabbits Undergoing Median Sternotomy

**DOI:** 10.3390/vetsci13090863

**Published:** 2026-08-26

**Authors:** Iulia Melega, Alexandra Creț, Maria-Carmen Turcu, Marcus Ofrim, Cosmin Petru Peștean, Lucia Victoria Bel

**Affiliations:** 1 Department of Surgery, Anesthesia and ICU, Faculty of Veterinary Medicine, University of Agricultural Sciences and Veterinary Medicine Cluj-Napoca, 400372 Cluj-Napoca, Romania; alexandra.cret@usamvcluj.ro (A.C.); cosmin.pestean@usamvcluj.ro (C.P.P.); 2 New Companion Animals Clinic, Department of Surgery and ICU, Faculty of Veterinary Medicine, University of Agricultural Sciences and Veterinary Medicine Cluj-Napoca, 400372 Cluj-Napoca, Romania; maria-carmen.turcu@usamvcluj.ro (M.-C.T.); alexandru-marcus.ofrim@student.usamvcluj.ro (M.O.); lucia.bel@usamvcluj.ro (L.V.B.)

**Keywords:** fascial plane anesthesia in rabbit, transversus thoracic plane, sternotomy, mediastinal mass

## Abstract

Two client-owned Lionhead rabbits underwent median sternotomy for mediastinal mass excision. Premedication included methadone, dexmedetomidine, and midazolam, with ketamine added for one rabbit. Anesthesia was maintained with isoflurane in oxygen and a ketamine infusion. Bilateral ultrasound-guided TTP blocks were performed at the level of the second and fifth intercostal spaces using a linear transducer and a spinal needle advanced in-plane in a lateromedial direction. Bupivacaine 0.5% was injected at a total dose of 2 mg/kg per rabbit, divided equally among four injection sites (total volumes of 0.9 mL and 1.0 mL for the first and second rabbits, respectively). Only one rabbit exhibited a brief intraoperative nociceptive response. The second rabbit developed severe postoperative respiratory compromise related to inadequate thoracic drain function and died 4 h after surgery. These cases indicate that the TTP block is technically feasible in rabbits and can contribute to intraoperative analgesia during thoracic surgery. Further studies are needed to evaluate the optimal injectate volume and its distribution, as well as the safety and efficacy of the anesthetic dose.

## Simple Summary

Thoracic surgery in rabbits carries a high risk of perioperative complications and pain when analgesia is inadequate. The transversus thoracic plane (TTP) block, a fascial plane technique aiming to provide ventral thoracic analgesia, has been previously described in dogs and cats, but not in rabbits. This report describes bilateral ultrasound-guided TTP blocks, modified from a feline approach at two intercostal spaces per side, in two rabbits undergoing median sternotomy for mediastinal mass excision. Bupivacaine 0.5% was administered at a total dose of 2 mg/kg per rabbit across four injection points. Both rabbits showed minimal intraoperative nociceptive responses, evidenced by stable heart rates and a low isoflurane requirement. Rabbit 1 recovered uneventfully and required rescue analgesia six hours postoperatively; rabbit 2 developed a complication unrelated to the block and died four hours postoperatively. To the authors’ knowledge, this is the first report describing the TTP block in rabbits, which appears feasible and contributes to intraoperative analgesia in thoracic surgery, but further study should determine the optimal injectate volume and distribution, as well as the safety and efficacy of the anesthetic dose.

## 1. Introduction

It is well known that pet rabbits have higher perioperative mortality rates than dogs and cats, with reported mortality rates of 0.73% [1] and 0.826% [2] in healthy rabbits, 7.14% [2] and 7.37% [1] in sick rabbits, and 1.39% [1] and 2.05% [2] overall, compared with 0.17–1.39% in dogs and cats [1]. Furthermore, perioperative pain management remains one of the major challenges in rabbit anesthesia, owing to the species’ particular pharmacokinetic features, in addition to the high risk of complications associated with the long-term use of systemic analgesics such as opioids [2]. Despite the high number of rabbits undergoing surgical procedures every year [3], anesthesia and analgesia protocols for rabbits remain limited compared with those for cats and dogs.

Thoracic procedures are considered high-risk, non-routine surgeries associated with an elevated risk of mortality [4]. The most common indication for thoracic surgery in companion rabbits is the excision of mediastinal masses, such as thymomas, lymphomas, or lung abscesses [5,6]. Surgeries involving the chest wall are frequently associated with intraoperative nociceptive responses to surgical stimulation and significant postoperative pain [7]. The main complication following median sternotomy for thymoma removal in rabbits is acute perioperative death, which may be associated with pain, stress, anesthetic complications, or incomplete tumor excision, with a notably high mortality rate within the first three days [5]. Given the physiological and behavioral characteristics of rabbits as prey species, optimal perioperative analgesia is essential [5]. Consequently, balanced anesthetic techniques incorporating regional anesthesia are expected to offer important advantages for procedures involving this region of the body, as demonstrated in human patients [7].

Ensuring effective analgesia for major thoracic surgeries is one of the most challenging tasks for anesthetists [7]. Intercostal nerve blocks are commonly used to provide perioperative analgesia in small animals undergoing lateral thoracotomies and, when performed bilaterally across multiple intercostal spaces, for median sternotomy as well [8]. However, the success rate of intercostal nerve blocks is lower in rabbits than in dogs [9].

A technique that has recently gained attention is the transversus thoracic plane (TTP) block. This block technique involves injecting a local anesthetic solution parasternal into an interfascial space, where the ventral cutaneous branches of the intercostal nerves lie between the transversus thoracis muscle and the internal intercostal muscles (*mm. intercostales interni*). When performed bilaterally, this approach aims to provide analgesia to the ventral aspect of the thorax by blocking the nerves responsible for sensory innervation of the sternum, midline skin, costal pleura, and related structures [10,11,12]. The technique has demonstrated utility in companion animals, enhancing perioperative care by improving the quality of intraoperative analgesia [13,14,15,16].

The aim of this two-case report was to describe the feasibility and advantages of the TTP block in rabbits as part of a multimodal analgesia protocol, extrapolating from methods previously reported in dogs and cats.

## 2. Case Presentation

### 2.1. Patients

Two unrelated pet rabbits were presented to the New Companion Animal Clinic, Faculty of Veterinary Medicine, Cluj-Napoca, in 2024 and 2025 for evaluation of suspected cranial mediastinal masses. Both owners were informed about the risks of the procedures and signed the standard clinical consent form used in routine veterinary practice. At the initial evaluation, both rabbits underwent a full clinical examination, full-body radiographs, complete blood count (CBC) and biochemistry (data available upon request). Both owners were offered IgG and IgM testing for *E. cuniculi* but refused due to financial constraints.

Rabbit 1: A 2.2 kg, 5-year-old spayed female Lionhead rabbit was presented with a history of decreased appetite, an abdominal breathing pattern, respiratory distress, and muffled heart sounds on auscultation. The rabbit also exhibited moderate exophthalmos and a left head tilt, consistent with chronic otitis media. Serum biochemistry revealed hypoalbuminemia (1.6 g/dL; reference range 2.8–4 g/dL), elevated ALT (102 U/L; reference range 52–80 U/L) and hyperkalemia (6.2 mEq/L; reference range 3.5–5.1 mEq/L). Complete blood count showed leukocytosis (WBC 13.15 × 10^9^/L, reference range 3–12.5 × 10^9^/L) with neutrophilia (8.15 × 10^9^/L, reference range 1.02–5.95 × 10^9^/L). Abdominal radiographs were unremarkable, while thoracic radiographs revealed a mass within the cranial mediastinum. Under deep sedation using an intramuscular combination of medetomidine (0.05 mg/kg), ketamine (5 mg/kg), and midazolam (0.5 mg/kg), together with continuous oxygen therapy using a facial mask, cranial and thoracic computed tomography was performed to further characterize the lesion. The examination confirmed a non-contrast-enhancing mass within the cranial mediastinum and concurrent left-sided otitis media. Ultrasound-guided fine needle aspiration (FNA) was refused due to the risk of hemorrhage. Because a 2-week course of prednisolone offered no benefit and clinical signs worsened, exploratory median sternotomy was elected to assess and eventually remove the thoracic mass.

Rabbit 2: A 2.4 kg, 5-year-old castrated male Lionhead rabbit was initially presented to a referring veterinary service for routine vaccination. During the pre-vaccination physical examination, muffled heart sounds were detected on thoracic auscultation, prompting further diagnostic investigation. Full-body radiography was performed. While abdominal imaging was normal, thoracic radiographs revealed a cranial mediastinal mass, and cytologic examination of an FNA from the mass raised suspicion of a mediastinal abscess. Serum biochemistry showed hypoalbuminemia (1.4 g/dL), while CBC was unremarkable. The rabbit was subsequently referred to the New Companion Animal Clinic for further evaluation and treatment. Although additional advanced imaging was considered during the diagnostic work-up, it was declined due to financial constraints. As in rabbit 1, exploratory median sternotomy was suggested and approved by the owner as the definitive diagnostic and potentially therapeutic approach.

### 2.2. Anesthesia Protocol

At physical examination before anesthesia, both rabbits showed increased respiratory effort, with a respiratory rate of 60–80 breaths/min and shallow breathing. Mucous membranes could not be reliably evaluated in rabbit 1 due to its behavior, whereas they were pink and moist in rabbit 2. Heart rate (HR) could not be reliably counted by auscultation in both animals due to the presence of the cranial thoracic mass; however, cardiac rhythm was assessed to be regular, with no arrhythmia detected on pulse palpation.

Both rabbits were assigned an American Society of Anesthesiologists physical status classification of 3/5 and received the same premedication protocol, administered as a single intramuscular injection into the epaxial muscles. The protocol consisted of methadone (0.4 mg/kg), dexmedetomidine (0.025 mg/kg), and midazolam (0.5 mg/kg). For rabbit 1, ketamine (3 mg/kg) was additionally included in the premedication mixture to increase the depth of sedation given her anxious behavior. Following an appropriate level of sedation, two intravenous catheters were placed for each rabbit: one in the marginal auricular vein and the second in either the cephalic antebrachial vein (rabbit 1) or the saphenous vein (rabbit 2).

In rabbit 1, sedation resulted in respiratory compromise, which required rapid induction with intravenous (IV) propofol (2 mg/kg); given the urgency of the situation, topical lidocaine was not administered prior to intubation. Orotracheal intubation was performed using a compact endoscope (1.9 mm, Eickemeyer, Tuttlingen, Germany) with a 3.5 mm internal diameter uncuffed endotracheal tube, and manual ventilation was initiated via a T-piece non-rebreathing circuit. During this period, HR could not be reliably captured given the emergent focus on respiratory support; once stabilized, HR was in the upper 170 beats per minute (bpm) range. In rabbit 2, preoxygenation was carried out for five minutes using an oxygen cage prior to induction. Anesthesia was induced with ketamine (3 mg/kg IV), and intubation was facilitated by topical application of lidocaine 2% on the larynx. A 3.0 mm internal diameter uncuffed endotracheal tube was placed using the same technique as described for the first rabbit. At this stage, the HR was 144 bpm (135–154 bpm), and the respiratory rate was 68 breaths/min, decreasing to 12 breaths/min after 30 min. In both rabbits, anesthesia was maintained with isoflurane in O_2_, delivered via a T-piece non-rebreathing circuit during surgical preparation and a pediatric rebreathing circuit throughout the surgical procedure. The vaporizer was initially set at 1.5% during surgical preparation and gradually decreased after completion of the TTP blocks.

Continuous intraoperative monitoring was performed in both rabbits using a multiparameter monitor (Mindray ePM 12M Vet, Mindray Animal Medical, Shenzhen, China), with parameters including electrocardiography (ECG) for HR, pulse oximetry (SpO_2_) for peripheral oxygen saturation and pulse rate, end-tidal carbon dioxide (EtCO_2_), respiratory rate (fR), tidal volume (V_t_), esophageal temperature, and the expired fraction of isoflurane (E_t_Iso) until recovery. Anesthetic depth was assessed based on muscle tone, palpebral and corneal reflexes, and the presence of spontaneous movement. Analgesia was considered inadequate in the presence of autonomic responses to surgical stimulation, defined as an increase in HR of >20% from baseline, accompanied or not by spontaneous movement and/or changes in ventilation parameters. In Rabbit 1, arterial catheterization for invasive blood pressure monitoring was not pursued because the rabbit became unstable, while in Rabbit 2, invasive blood pressure monitoring was not attempted. Oscillometric non-invasive monitoring was performed in both cases; however, values showed variability between consecutive readings and were therefore not considered valid for reporting. Intravenous fluid therapy was maintained using Ringer’s solution at an initial rate of 10 mL/kg/h, with the infusion rate adjusted as clinically indicated during the procedure.

### 2.3. Transversus Thoracic Plane Block

After anesthesia induction, the rabbits were positioned in dorsal recumbency, the surgical site was clipped, and the skin was scrubbed using chlorhexidine 2% (Lifo-Scrub, B. Braun Medical AG, Sempach, Switzerland) and alcohol 70%.

**Figure 1:**
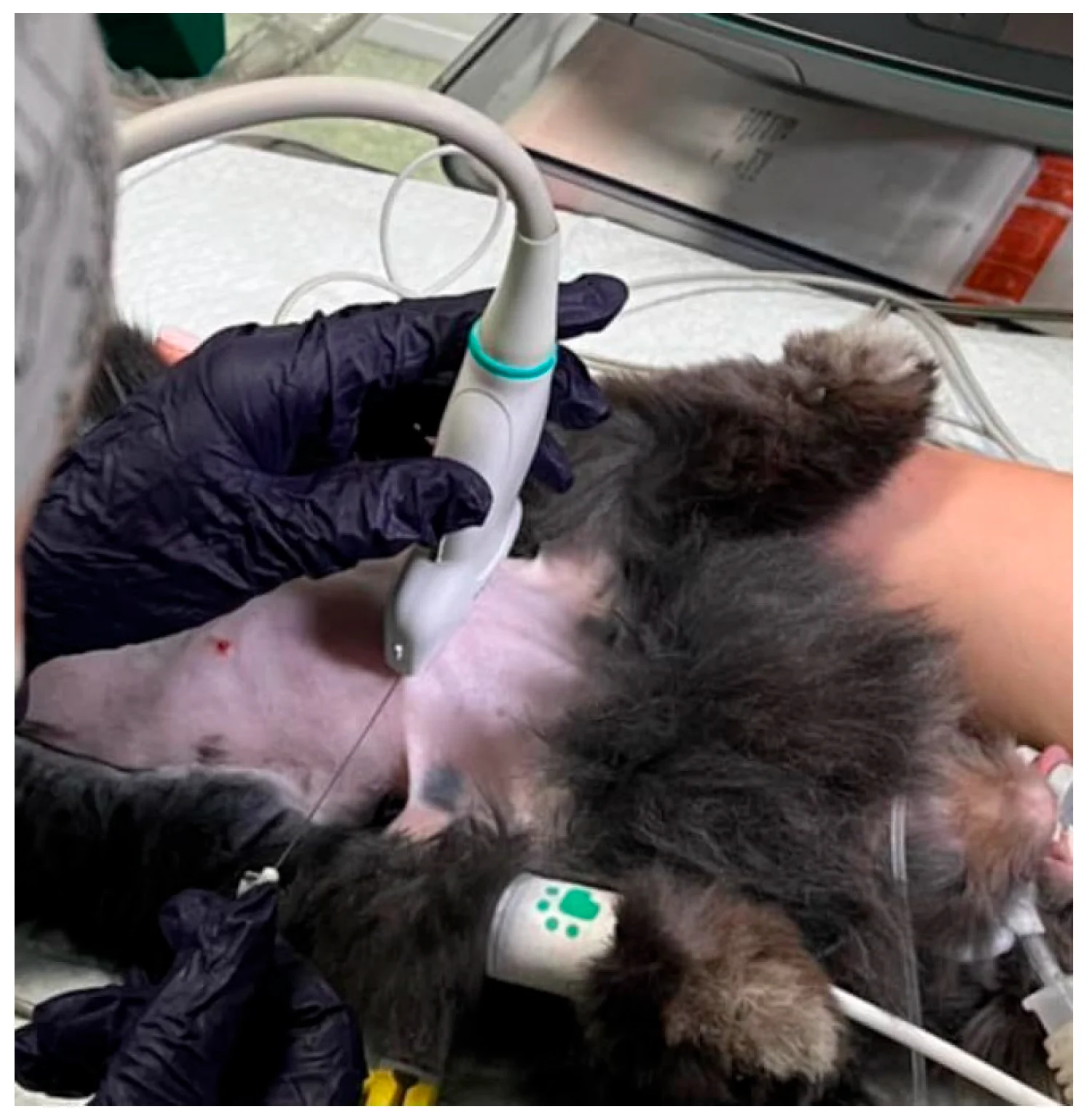
Ultrasound-guided TTP block in the rabbit. The ultrasound probe is positioned perpendicular to the sternum, while the spinal needle is advanced in-plane in a lateromedial direction at the level of the second intercostal space.

**Figure 2:**
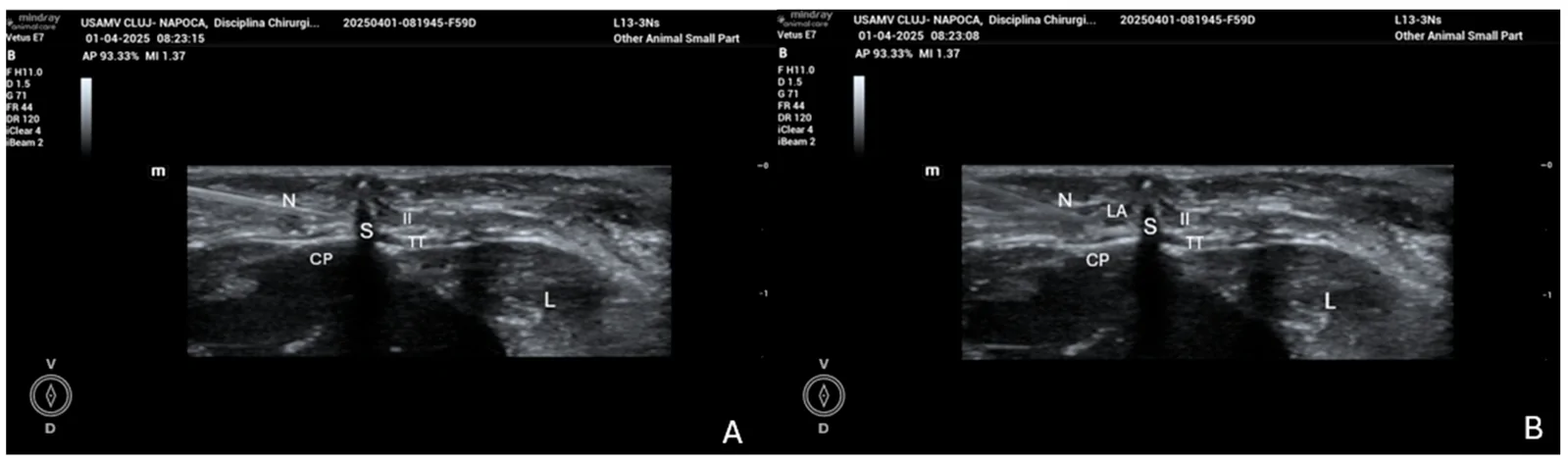
Sonoanatomy of the fifth intercostal space in the rabbit over the sternum. Ultrasound image showing the anatomical structures relevant to the transversus thoracis plane (TTP) block with the needle advanced toward the injection point, before the injection (**A**) and after the injection of local anesthetic (**B**). N, needle; CP, costal pleura; II, internal intercostal muscle; TT, transversus thoracis muscle; L, collapsed lung; LA, local anesthetic; S, sternum; V, ventral; D, dorsal.

Maintaining the rabbits in dorsal recumbency, the TTP block was performed by placing a high-frequency linear transducer perpendicular to the sternum at the level of the fifth intercostal space (Figure 1). The interfascial plane between the transversus thoracic and the internal intercostal muscles was sonographically identified (Figure 2A). Vascular structures were confirmed using color Doppler, and the internal thoracic vessels were taken as the principal anatomical landmarks. A 22 G, 75 mm spinal needle (Spinal needle, Vetro Medical Instruments, Bucharest, Romania) was then advanced in-plane in a latero-medial direction into the target location. If the sternebra was touched, the needle was withdrawn a few millimeters. The local anesthetic was slowly administered, and a small pocket of fluid was visualized (Figure 2B). After completing the injection, the needle was withdrawn, and the block was repeated at the level of the second intercostal space. After completion on the first hemithorax, the same technique was performed on the second hemithorax.

Bupivacaine 0.5% was used at a total dose of 2 mg/kg per rabbit, divided equally across four injection points, with total injected volumes of 0.9 mL and 1 mL for the first and second rabbits, respectively. All blocks were performed by the same operator.

#### 2.3.1. Intraoperative Period

Following the last TTP block, the rabbits were transferred to the operating theater and connected to the breathing system delivering vaporized isoflurane in oxygen and medical air (FiO_2_ 0.8). Before mechanical ventilation was initiated in rabbit 1, progressive bradycardia (HR nadir 112 bpm, from a baseline of 170 bpm) and hypoxia (SpO_2_ 87%) were observed; these gradually resolved following IV atropine administration (0.05 mg/kg) and initiation of mechanical ventilation, with HR increasing to a peak of 180 bpm.

In both rabbits, pressure-controlled ventilation was used, with an initial peak inspiratory pressure of 10 cmH_2_O set to deliver a tidal volume of 6–8 mL/kg (Mindray WATO EX-65 PRO VET, Mindray Animal Medical, China); during open chest surgery, this was gradually increased to 14 cmH_2_O with a positive end-expiratory pressure of 3–5 cmH_2_O. Respiratory rate was adjusted to maintain an EtCO_2_ of 30–40 mmHg, and body temperature was supported using a heating mat. Surgery began approximately 30 min after TTP block in both animals.

Both rabbits underwent a ventral median sternotomy to provide bilateral access to the cranial mediastinum. As part of the multimodal analgesia protocol, a constant rate infusion (CRI) of ketamine at 0.01 mg/kg/min was started prior to surgical incision. A midline skin incision was made extending from the cervical region to the xiphoid process, followed by dissection of the pectoral and sternohyoid muscles. The sternebrae were then divided along the midline using an oscillating sagittal saw, and a thoracic retractor was placed to improve exposure of the surgical field.

In rabbit 1, E_t_Iso was maintained at 1–1.1% throughout the procedure, and HR remained stable at 165 bpm (range, 154–180 bpm). Complete excision of the mediastinal mass was achieved, although firm adhesions between the mass and the adjacent lung parenchyma necessitated a partial lung lobectomy. No intraoperative complications were observed.

In rabbit 2, E_t_Iso was maintained at 1–1.1%, while HR progressively increased from 152 to 176 bpm during sternotomy and pneumothorax occurrence. As this increase remained less than 20% from baseline, no additional analgesia was administered. Approximately 50 min after the TTP block, during the initial manipulation of the mass, HR increased gradually to 180 and thereafter to 220 bpm (22% increase). No changes in EtCO_2_ or ventilation pattern were observed, and the episode was successfully managed with intravenous ketamine (0.5 mg/kg). Complete excision was not feasible because of extensive involvement of the surrounding structures, and partial excision of the mediastinal mass was therefore performed.

A 6 Fr double-lumen central venous catheter (ARROW^®^, Teleflex, Wayne, PA, USA) was placed in the 8th intercostal space in both rabbits as a thoracic drain to actively evacuate residual air and fluid from the thoracic cavity and to allow injection of local anesthetic for postoperative regional analgesia. In rabbit 2, closure of the thoracic cavity resulted in severe respiratory compromise: despite confirmed negative pressure on aspiration, progressive hypoxia and tachycardia followed by bradycardia developed, with low tidal volumes achieved during mechanical ventilation, suggesting inadequate ventilation or tension pneumothorax as the primary cause. Mechanical ventilation was stopped, intravenous atropine (0.05 mg/kg) was administered, and repositioning the animal from dorsal to lateral recumbency restored drain patency. Drain aspiration of an additional 80 mL of air led to transient improvement in respiratory function and SpO_2_; however, cardiovascular and respiratory function deteriorated gradually in the immediate postoperative period, and the rabbit died four hours later.

Surgical procedure durations were 105 and 80 min for the first and second rabbits, respectively, with total anesthesia times of 160 and 140 min.

#### 2.3.2. Postoperative Period

Rabbit 1 was extubated 18 min after the end of anesthesia. Body temperature at this time was 35.6 °C, and SpO_2_ had decreased to 86%, necessitating supplemental oxygen therapy; meloxicam (1 mg/kg SC q24h) and metamizole (30 mg/kg IM q12h) were administered, and the rabbit was placed in an incubator to provide oxygen supplementation and a warm environment. Postoperative pain was assessed using the Rabbit Pain Behavior Scale [17] six hours after the fascial block was performed; a fentanyl–lidocaine–ketamine (FLK 0.1–20-2 µg/kg/min, 10 mL/kg/hr) CRI was initiated in response to a pain score of 9/12, exceeding the intervention threshold of >3/12. Additional locoregional analgesia was provided by instillation of bupivacaine 0.5% (1 mg/kg) through the thoracic drain every eight hours; further pain scoring was precluded by personnel limitations. Supportive care included assisted feeding, subcutaneous antibiotic therapy with marbofloxacin (2 mg/kg q24h), and thoracic drain aspiration every four hours. The following day, the thoracic drain was removed, and the FLK infusion was replaced with buprenorphine (0.05 mg/kg q8h IV) combined with a lidocaine infusion (0.02 mg/kg/min). The rabbit was discharged 6 days after the surgical procedure.

Pain assessment for the second rabbit was not performed due to the critical postoperative complications described above.

Histopathological examination of the excised masses confirmed a diagnosis of thymic lymphoma in rabbit 1 and thymoma in rabbit 2. In the surviving rabbit, recurrence was detected during the five-month follow-up period, and chemotherapy with lomustine was initiated, extending survival time by a further two months.

## 3. Discussion

Complex surgical procedures in pet rabbits have gained popularity due to the growing compliance of owners with medical recommendations and the increasing level of care provided by their owners. Intrathoracic and mediastinal masses in pet rabbits are dominated by thymoma, with thymic lymphoma being less common. Due to high mortality rates, corticosteroids, chemotherapy and radiation therapy are presented as better alternative therapeutic options to surgery. Medical management with prednisolone monotherapy has emerged as a notably effective, low-cost, and low-risk option: a retrospective comparison of 33 rabbits found that median survival was longest in the prednisolone monotherapy group at 270 days, compared with 213 days for radiation therapy and 92 days for untreated rabbits, and follow-up analysis confirmed that prednisolone alone may significantly extend survival time and improve quality of life in rabbits with thymoma, offering an additional low-cost, effective option with minimal side effects [18]. Radiation therapy has also produced meaningful clinical improvement [6]. When radiation therapy is not available, fine needle aspiration is inconclusive as a basis for treatment or if the animal does not respond to steroid therapy, as in our cases, surgery could be taken into consideration. Consequently, the development of safe and effective anesthetic techniques has become essential.

The locoregional anesthesia techniques most routinely performed in rabbits are intratesticular injections and local infiltration [19]; however, peripheral nerve blocks and fascial plane blocks have received increasing interest in recent years [20,21]. As locoregional anesthesia has become part of the standard of care in dogs and cats [22], this has provided the motivation to develop equivalent techniques in rabbits, translating approaches established in companion animals and human medicine.

Thoracic surgery is associated with severe perioperative pain if insufficient analgesia is provided. In pediatric patients undergoing cardiac surgery, adequate pain control is a critical component of perioperative care. Uncontrolled pain can precipitate serious hemodynamic and respiratory complications with downstream consequences of increased morbidity and an extended length of admission period [23].

The ultrasound-guided transversus thoracic plane block is a newly evolving method that was first described for human patients in 2015 by Ueshima and Kitamura [11], and this approach has recently been extrapolated for use in dogs [14,15,24] and cats [13,14].

Two ultrasound-guided approaches to the TTP block have been described in dogs, differing in probe orientation: a sagittal approach [25] and a transverse approach [10,12,15], both evaluated through cadaveric studies assessing injectate spread. The most consistent and extensive dye distribution was achieved using a two-point injection technique at the third and sixth intercostal spaces with a high injectate volume (1 mL/kg), advancing the needle in a ventro-medial to dorso-lateral direction using an in-plane technique [12]. When applied in vivo, this technique was deemed more reliable for intraoperative and postoperative analgesia compared with the one-point injection technique, where concomitant intraoperative infusion of lidocaine and ketamine has been used [15].

A modified single-point approach at the fourth intercostal space, with the needle directed dorsolaterally to ventromedially, was described by Bartolome Rico Pérez et al. (2023) in a cat undergoing sternotomy for mediastinal mass removal—a modification motivated primarily by the patient’s small size and the need to optimize needle visualization [16]. The present report adopted the same modified approach; however, local anesthetic was administered at two injection points rather than one, with the aim of achieving broader injectate distribution. The selection of the second and fifth intercostal spaces in the present report, rather than the third and sixth spaces described in canine cadaveric studies [12], may be justified by the comparatively shorter and more compact rabbit thorax: the rabbit sternum comprises only six sternebrae, corresponding to seven pairs of sternal ribs [26]. Based on this, the authors considered that the craniocaudal span likely necessitates a more cranially shifted injection site to achieve equivalent nerve coverage relative to larger species. In contrast, Degani et al. employed the parasagittal approach described by Alaman et al. [10], combining smaller volumes with a bilateral six-point injection technique spanning the second to seventh intercostal spaces in a feline patient undergoing sternotomy for lung lobectomy, as the authors argued that anesthetic spread in cats could not be assumed to resemble that observed in dogs [13]. Compared with their technique, the authors of the present report considered this approach more challenging because of the small patient size, with a potentially higher risk of complications and a longer procedure time.

In the present report, both blocks appeared to improve analgesia based on the minimal response to pain recorded during the surgery. Loco-regional anesthesia is considered effective if it produces adequate sensory blockade of the targeted area, as demonstrated by the absence of a response to noxious stimulation, attenuation of intraoperative cardiovascular responses, and a reduction in systemic anesthetic and analgesic requirements [22,27]. The rabbits in the present report were anesthetized using a balanced anesthesia protocol, with continuous monitoring of vital parameters and anesthetic requirements throughout the surgical procedure. It should be noted that the reduction in anesthetic requirement observed in the present cases cannot be attributed to the loco-regional block in isolation; multimodal analgesia—through the additive or synergistic effects of systemically administered analgesics such as opioids and NMDA-receptor antagonists—is well recognized to produce an MAC-sparing effect independent of any regional technique [3,28].

Cardiovascular variables such as HR and blood pressure and ventilatory variables like fR and V_t_ have been successfully associated with responses to surgical stimulation in humans [29] and dogs [30], while anesthetic depth alone is considered to have limited utility for this purpose [29]. In anesthetized rabbits undergoing stifle arthrotomy, invasive blood pressure monitoring has been shown to be an imperfect surrogate for intraoperative nociception, with low sensitivity and specificity, and its use requires dedicated pressure transducers and arterial catheterization, which are not always readily available in clinical settings [31]. The Doppler method has demonstrated good correlation with invasively measured systolic blood pressure values and is considered suitable for monitoring systolic pressure [32]; however, it was not used in the present cases, as dorsal recumbency during sternotomy limited access to the distal extremities typically used for Doppler probe placement, and the surgical drapes further restricted intraoperative access to these sites. Non-invasive oscillometric blood pressure monitoring was therefore selected for cardiovascular assessment in the present cases; however, readings were inconsistent and were considered unreliable by the authors.

Mechanical ventilation was maintained throughout the surgical procedure in both rabbits; therefore, fR and V_t_ could not be used as indicators of intraoperative nociceptive responses. Instead, intraoperative nociception was assessed primarily based on HR and anesthetic depth. Only one of the two rabbits (rabbit 2) required a single rescue bolus of ketamine, administered 50 min after block performance in response to apparent nociceptive stimulation, in addition to the continuous ketamine infusion maintained throughout the procedure. Both rabbits received atropine for the treatment of bradycardia; however, in the first rabbit, bradycardia occurred before the onset of surgical stimulation, whereas in the second rabbit, it was associated with the acute respiratory compromise secondary to tension pneumothorax.

Regarding anesthetic depth, an E_t_Iso of 1.1% recorded in both rabbits represents approximately 0.6 minimum alveolar concentration (MAC), based on the baseline MAC of 1.83 ± 0.12% atm reported for New Zealand White rabbits by Barter & Pypendop (2025) [28]. This is well below the threshold generally required for reliable surgical tolerance with isoflurane alone. Ketamine, an anesthetic agent with analgesic properties acting primarily at NMDA receptors, was concurrently administered as a CRI in both rabbits. Ketamine has previously been shown to reduce isoflurane MAC in rabbits by 24% following a loading dose of 1 mg/kg with a CRI of 40 µg/kg/min [33]. More recently, Barter & Pypendop (2025) [28] demonstrated that the relationship between plasma ketamine concentration and isoflurane MAC reduction in rabbits is non-linear, with MAC reductions of approximately 24, 36, 44, 53, and 61% at plasma concentrations of 1, 2, 4, 8, and 12 µg/mL, respectively. Given this non-linear relationship, the contribution of the lower-dose ketamine CRI used in the present cases to overall MAC reduction cannot be precisely extrapolated from higher-dose studies; nonetheless, based on the reported concentration–effect curve, an important but likely submaximal reduction in isoflurane requirement would be expected at the infusion rate used here. The absence of a significant intraoperative nociceptive response was therefore most likely attributable to the combined contribution of the multi-modal anesthesia protocol and the loco-regional anesthetic technique, rather than to either component alone, as discussed above.

The efficacy of the block during the postoperative period remains questionable, based on the pain score recorded six hours after block performance. Earlier evaluation was not possible due to logistical constraints related to postoperative monitoring requirements, which unfortunately delayed both pain recognition and appropriate treatment. Furthermore, postoperative assessment was limited to one rabbit, as the critical condition of the second animal precluded reliable use of the pain scale. The duration of sensory blockade achieved with bupivacaine in rabbits varies depending on the anatomical site, drug concentration, and specific technique employed. Successful auricular nerve blocks have yielded a mean sensory block duration of 88 ± 52 min in conscious rabbits [34], while significant sensory blockade following sciatic nerve block resolved at approximately 120 min post-injection [35]. When bupivacaine was combined with lidocaine 2%, postoperative rescue analgesia was required in 25% of rabbits within the six-hour postoperative period following sciatic nerve block [31]. It is important to recognize that the TTP block is a fascial plane block rather than a targeted peripheral nerve block. Therefore, this technique should not be expected to achieve the same degree or duration of neural blockade as a peripheral nerve block, where the volume of local anesthetic is deposited in close proximity to the target nerves. Achieving a comparable effect with a fascial plane block may require a correspondingly larger injectate volume [36].

Regional anesthesia is not limited to the intraoperative period; local anesthetics can also be administered on an intermittent basis during recovery through the use of interpleural catheters [37]. Previous evidence indicates that with a single injection, the local anesthetic solution spreads to the intercostal nerves, providing a safe and effective modality for improving pain control following thoracotomy [37,38]. When evaluated in post-sternotomy dogs, interpleural bupivacaine was associated with higher cortisol levels compared with interpleural or intramuscular morphine, yet yielded similar pain scores—suggesting that the technique may offer meaningful benefit as a complement to systemic analgesic administration [39]. In the present study, bupivacaine 0.5% was administered at a dose of 1 mg/kg every 8 h. The choice of administration frequency was intended to limit the risk of cummultive effects associated with interpleural drug administration, as well as to accommodate personnel availability. No studies are currently available regarding the effectiveness, pharmacokinetics, or complications of interpleural bupivacaine in rabbits; therefore, the authors followed the protocol described for repeated bolus injections of bupivacaine for continuous bilateral hemithorax analgesia in a cat [40].

Potential complications associated with TTP block include accidental intravascular injection and inadvertent puncture of the thoracic pleura. As the interfascial plane lies relatively deep and in close proximity to the pulmonary pleura, particular care is required, especially in small patients such as rabbits. An alternative to TTP is the ultrasound-guided pecto-intercostal fascial plane block, performed between the deep pectoral and external intercostal muscles [41], which carries a lower risk profile owing to its more superficial location relative to the thoracic cavity. This technique targets the ventral cutaneous branches of the intercostal nerves and has been successfully employed in four dogs to provide intraoperative analgesia during sternotomy, making it a promising substitute for the TTP block in this surgical context [42].

Fascial plane blocks require sufficient injectate volumes to ensure adequate distribution and efficacy; however, excessive doses carry the risk of local anesthetic systemic toxicity (LAST). In both rabbits, the total dose of bupivacaine 0.5% did not exceed 2 mg/kg, a dose commonly accepted for locoregional anesthesia in mammals [27]. Although this represents a conservative volume, it was intentionally chosen to reduce the risks associated with accidental intravascular and intrathoracic injection, given the close proximity of the injection sites to major vascular structures and the rapid absorption of local anesthetics.

No signs of toxicity were observed in either patient. Local anesthetic toxicity (LAST) can be difficult to diagnose in anesthetized patients, as classical clinical signs may be masked by general anesthesia. In the present report, the authors attributed the complication observed in rabbit 2 to drain malfunction rather than LAST: the rabbit developed impaired ventilation despite mechanical ventilation not having been discontinued, followed by hypoxia and, ultimately, bradycardia. Clinical signs improved following additional drain aspiration and atropine administration, supporting the presumptive diagnosis of tension pneumothorax.

Several limitations must be acknowledged in this report. First, the lack of blood pressure monitoring precluded a complete assessment of the nociceptive experience, as analgesia was evaluated using a limited set of parameters. Second, the small number of patients represents an important limitation. With only two rabbits, it is not possible to fully confirm the efficacy and safety of the technique within a multimodal anesthesia context. Third, the timing of postoperative pain scoring was delayed, which may have missed the window in which earlier analgesic intervention was required, potentially underestimating the true extent of the patients’ pain. Finally, the fatality reported in rabbit 2 limited postoperative evaluation to a single patient.

## 4. Conclusions

To the authors’ knowledge, this is the first report describing the use of the TTP block in rabbits, applying the ultrasonographic landmarks previously established in companion animals. These preliminary findings suggest that the technique is feasible to perform and may add value in enhancing intraoperative analgesia; however, further clinical studies involving larger numbers of rabbits are required to better characterize its efficacy and safety profile.

This report contributes to the scarce literature on locoregional anesthesia techniques in exotic companion animals and highlights the need for continued investigation. Future studies should focus on characterizing injectate distribution and determining the optimal injection volume for this species, as well as evaluating the safety and efficacy of the anesthetic dose administered.

## Data Availability

The original contributions presented in this study are included in the article. Further inquiries can be directed to the corresponding author(s).
